# Pore size is a critical parameter for obtaining sustained protein release from electrochemically synthesized mesoporous silicon microparticles

**DOI:** 10.7717/peerj.1277

**Published:** 2015-10-06

**Authors:** Ester L. Pastor, Elaine Reguera-Nuñez, Eugenia Matveeva, Marcos Garcia-Fuentes

**Affiliations:** 1 EM Silicon Nanotechnologies, S.L., Valencia, Spain; 2 Center for Research in Molecular Medicine and Chronic Diseases (CIMUS), Universidade de Santiago de Compostela, Santiago de Compostela, Spain

**Keywords:** Mesoporous silicon, Pore size, Controlled release, Microparticles, Protein delivery, Bone morphogenetic protein

## Abstract

Mesoporous silicon has become a material of high interest for drug delivery due to its outstanding internal surface area and inherent biodegradability. We have previously reported the preparation of mesoporous silicon microparticles (MS-MPs) synthesized by an advantageous electrochemical method, and showed that due to their inner structure they can adsorb proteins in amounts exceeding the mass of the carrier itself. Protein release from these MS-MPs showed low burst effect and fast delivery kinetics with complete release in a few hours. In this work, we explored if tailoring the size of the inner pores of the particles would retard the protein release process. To address this hypothesis, three new MS-MPs prototypes were prepared by electrochemical synthesis, and the resulting carriers were characterized for morphology, particle size, and pore structure. All MS-MP prototypes had 90 µm mean particle size, but depending on the current density applied for synthesis, pore size changed between 5 and 13 nm. The model protein *α*-chymotrypsinogen was loaded into MS-MPs by adsorption and solvent evaporation. In the subsequent release experiments, no burst release of the protein was detected for any prototype. However, prototypes with larger pores (>10 nm) reached 100% release in 24–48 h, whereas prototypes with small mesopores (<6 nm) still retained most of their cargo after 96 h. MS-MPs with ∼6 nm pores were loaded with the osteogenic factor BMP7, and sustained release of this protein for up to two weeks was achieved. In conclusion, our results confirm that tailoring pore size can modify protein release from MS-MPs, and that prototypes with potential therapeutic utility for regional delivery of osteogenic factors can be prepared by convenient techniques.

## Introduction

Mesoporous silicon (MS)-based materials are currently investigated in a variety of systems for drug delivery and tissue engineering applications (Anglin et al., 2008; Santos, 2014). Their main advantage lies on their outstanding surface area arising from the fine mesoporous structure that allows remarkable drug loadings to be achieved just by plain adsorption (Prestidge et al., 2008). MS is also biocompatible (Canham, 1995; Godin et al., 2008; Salonen et al., 2008), and degrades in the body to silicates (SiO_2_) (Canham, 1995; Salonen et al., 2008; Pastor et al., 2009) that are eliminated by renal excretion (Popplewell et al., 1998). Silicates have FDA GRAS status, and even safety margins for silica nanoparticles administered intravenously start to be established (Yu et al., 2013). Inspired by these properties, researchers have investigated silicon-based carriers in a variety of formats (i.e., scaffolds, microparticles, nanoparticles, etc.) for delivering hydrophobic and hydrophilic drugs (Anglin et al., 2008; Prestidge et al., 2008; Salonen et al., 2008). MS-based materials have also been proposed for delivering drug-loaded nanoparticles within the concept of multistage delivery vehicles (Tasciotti et al., 2008).

Devices composed of a crystalline mesoporous silicon matrix are alternatives to silica mesoporous structures (Kresge et al., 1992), but unlike those, they do not require mesophase template removal for their preparation. Mesoporous silicon can be prepared by stain-etching or electrochemical anodizing of silicon. Both methods result in suitable mesoporous (nanostructured) materials, but the stain-etching method is less controlled with respect to pore homogeneity, and often leaves an untreated crystalline silicon core inside the particles. Medical materials prepared from stain-etched mesoporous silicon should be additionally checked for complete removal of toxic nitric oxide residues. The electrochemical method for MS production is therefore more medical-friendly, and recently its scalability has been considerably improved (Makushok, Matveyeva & Pastor, 2012).

The desired nanostructure of MS fabricated by electrochemical methods can be easily achieved by a simple tuning of the preparation conditions; first of all, the applied current density. Even though these inner nanostructure parameters (pore size, overall porosity, particle size, etc.) are important for MS silicon drug carriers, they cannot assure by themselves optimal drug payloads. The interaction between the drug and the carrier surface needs also to be engineered, and thus the surface modification and functionalization of MS nanostructures has been extensively studied in recent years (Jarvis, Barnes & Prestidge, 2011; Jarvis, Barnes & Prestidge, 2012; Barnes, Jarvis & Prestidge, 2013). Among different techniques, a simple oxidation is frequently performed that converts the outer surfaces of crystalline mesoporous silicon to a mesoporous silica replica (Kresge et al., 1992).

In a previous publication from our group, MS microparticles (MS-MPs) with an average pore size of 35 nm were prepared by an electrochemical method and stabilized by thermal oxidation. These MS-MPs were successfully loaded by absorption equilibrium with two model proteins, insulin and bovine serum albumin BSA (Pastor et al., 2011). Although these proteins were released from a vehicle in a controlled manner, the process was fast (∼80–100% release in less than 2 h), and consequently only suitable for some applications such as mucosal drug delivery.

Previous studies with hydrogels (Peppas et al., 2000), solid polymers (Sandor et al., 2001), and other mesoporous materials (Santos, Radin & Ducheyne, 1999) have shown that modulation of the inner nanostructure of the carrier can change the kinetics of drug release. We proposed that similar principles should apply for controlling the release of proteins from electrochemically synthesized MS-MPs. To address this hypothesis, we prepared MS-MPs with different pore sizes and explored how changes in inner nanostructure can influence the release of loaded proteins. This study was performed initially with the model protein *α*-chymotrypsinogen (aCT); then, considering the bioactivity of MS materials for orthopedic regeneration (Canham, Reeves & Newey, 1999; Pastor et al., 2007; Sun et al., 2007), we loaded a protein of therapeutic interest for this application, bone morphogenetic protein-7 (BMP7).

## Materials and Methods

### Materials

Boron doped silicon with different resistivity, 0.01–0.02 and 10–20 Ω cm, was purchased from Si Materials (Germany); wafer diameter was 100.0 ± 0.5 mm and thickness of 525 ± 25 µm (*pI* = 2–3.5). Fluoric acid (HF) (48%) was purchased from Riedel de Haën (Germany) and ethanol (96%) from Panreac (Barcelona, Spain). Synthetic air (*N*_2_ with 21% of *O*_2_) was provided from AbelloLinde S.A. (Barcelona, Spain). Avidin-peroxidase conjugate, *α*-chymotrypsinogen A (aCT) from bovine pancreas (*pI* = 9.5; *Mw* = 25.7 kDa), and 2, 2′-azino-bis (3-ethylbenzthiazoline-6-sulfonic acid) were obtained from Sigma Aldrich (Madrid, Spain). Recombinant human Bone Morphogenetic Protein-7 (BMP7) (*pI* = 8.1; *Mw* = 28.8 kDa), polyclonal antibody rabbit anti-human BMP7, and biotinylated polyclonal antibody rabbit anti-human BMP7 were purchased from PeproTech (London, UK). All other solvents and chemicals used were high-grade purity.

### Preparation of mesoporous silicon microparticles (MS-MPs)

MS-MPs were obtained by an electrochemical method similar to that previously described by us (Pastor et al., 2009). The main difference was the use of a 1:1 HF:Ethanol electrolyte, and special cyclic regimes with etch-stops in order to improve the homogeneity of pore sizes distribution along with the in-depth etching (Bychto et al., 2008). A constant current step (40 or 60 mA/cm^2^ for 5–10 s) was followed by an etch-stop step (no current applied for 2–5 s) in cyclic periods. After obtaining a MS layer of ∼150 µm thickness, the electrochemical process was stopped, and the Si wafer was washed thoroughly with distilled water, dried, and the porous material was scratched from the remaining Si substrate. The obtained MS was subjected to a thermal oxidation under a flow of synthetic air for 1 h at 500 or 650 °C (Programat P200 equipped with a vacuum pump VP3 and gas inlet; Ivoclar-Vivadent, Inc., Amherst, New York, US). To reduce the particle size to the micrometer scale, the MS material was milled and sieved in cascade. The fraction between 75 and 100 µm was selected for further studies. Henceforth, this fraction is referred to as MS-MPs. The preparation conditions for the three different MS-MP prototypes studied in this work are summarized in Table 1. For example, prototype B was prepared from Si wafer of 0.01–0.02 Ω cm resistivity, under a current density of 40 mA/cm^2^ applied for 10 s, and then interrupted by a 2 s interval of zero current (etch-stop). This regime was cyclically repeated for a few hours until the 150 µm porous layer was grown. After recollecting the porous material, the material was thermally oxidized at 650 for one hour.

**Table 1:** Preparation conditions for different mesoporous silicon prototypes synthesized by the electrochemical method under special cyclic regimes with etch-stop (zero current) applied after each anodizing interval. Three different prototypes (A–C) were prepared and tested in this study, differing in silicon waver resistivity, current densities, etch-stop times, and thermal oxidation temperatures.

Prototype	Si wafer resistivity (Ω cm)	Current density (mA/cm^2^)/ anodizing time (s)	Etch stop time (s)	Oxidation temperature (°C)
A	0.01–0.02	40/5	5	500
B	0.01–0.02	40/10	2	650
C	10–20	60/5	2	550

### Characterization of MS-MPs

The porosity of the porous silicon materials was determined gravimetrically by comparing the mass of the silicon wafer before and after anodizing as previously described (Pastor et al., 2011). Particle sizes were analyzed with a Mastersizer 2000 (Malvern Instruments, Malvern, Worcestershire, UK). MS-MPs morphology was visualized by high resolution Scanning Electron Microscopy (SEM; Hitachi S4500; Hitachi, Tokyo, Japan). Additionally, the Brunauer- Emmett-Teller (BET) surface area of the MS-MPs was determined by *N*_2_ adsorption–desorption isotherms (Micrometrics ASAP 2020 V3.04H; Micromeritics France S.A.,Verneuil-en-Halatte, France). Pore size was calculated from the same *N*_2_ adsorption data, by the Barroett-Joyner-Halenda (BJH) method.

### Protein loading

Protein loading was carried out by solvent evaporation (Prestidge et al., 2008). Briefly, 20 µL of the model protein aCT (3 mg/mL) or BMP7 (5 µg/mL) in aqueous solutions were added to a fixed amount of MS-MPs (1 mg). The samples were gently vortexed for 10 s, and then incubated under mild agitation at 37 °C until total evaporation of solvent was reached and all amounts of proteins incorporated into the MS-MPs (about 7 h). The theoretical protein loadings were: 60 µg/mg of MS-MPs for aCT, and 0.1 µg/mg of MS-MPs for BMP7. Loaded MS-MPs were freeze-dried and stored at −20 °C until use.

### *In vitro* release studies

Samples comprising 1 mg of MS-MPs loaded with aCT or BMP7 were incubated with 500 µL of PBS (USP 38-NF 33, pH 7.4) under agitation (100 rpm, Titramax 1000; Heindolf, Schwabach, Germany) at 37 °C (Inkubator 1000, Heindolf, Schwabach, Germany). At scheduled time points, release samples were collected, and centrifuged at 7000 RCF for 10 min at 4 °C (Microfuge 22R; Beckman Coulter, Brea, California, USA). The amounts of aCT in supernatants were determined by the bicinchoninic acid method (Micro BCA protein Assay Kit; Pierce Biotechnology Inc., Rockford, Illinois, USA), and those of BMP7 by ELISA, as previously reported by us (Reguera-Nuñez et al., 2014). Amounts of released protein are expressed as percentage of a total protein mass added at the loading stage since the whole mass was considered as absorbed upon solvent evaporation.

## Results and Discussion

### Characterization of different MS-MPs carriers

**Figure 1:**
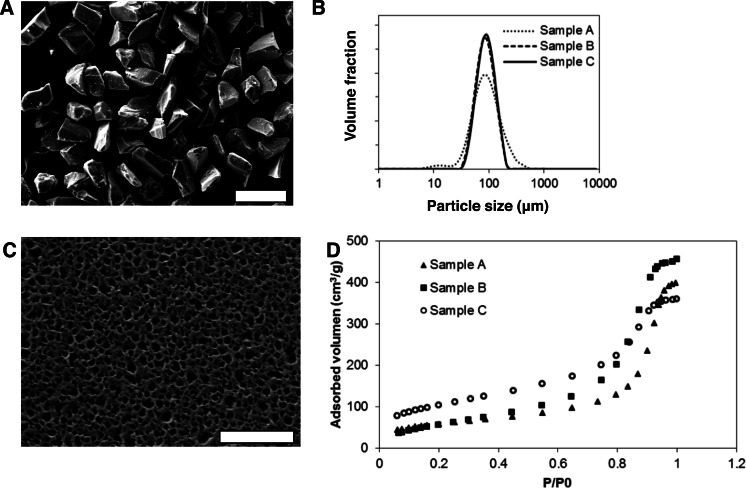
Morphological and physicochemical properties of mesoporous silicon microparticles (MS-MPs). (A) SEM image of MS-MPs (bar is 200 µm); (B) particle size distribution of the different MS-MP prototypes measured with a particle size analyzer; (C) example of a SEM image of the surface of MS-MPs (corresponding to prototype A, bar is 800 nm); (D) *N*_2_ adsorption isotherms, volume adsorbed vs. relative pressure (*P*/*P*0), for the different MS-MP prototypes.

Mesoporous silicon microparticles (MS-MPs) were prepared by electrochemical etching, thermal stabilization, and milling to reduce the particle sizes. The resulting powder was sorted by sieving. The particles of the selected fraction (i.e., the MS-MPs) were irregular in shape, but homogeneous in size (Fig. 1A). All the MS-MPs prototypes generated showed a normal distribution of sizes with a mean value around 90 µm (Fig. 1B). This normal particle distribution contrasted with our previous data where the particle distribution was log-normal (Pastor et al., 2011); this might be related to the different particle fractions selected on each work (90 µm vs. 33 µm mean size, respectively). The mesoporous structure of MS-MPs observed by high resolution SEM (Fig. 1C) revealed the regular and homogeneous pores propagated along a single direction, as it is common for electrochemically prepared MS. The SEM analysis, however, might not reveal the smallest pores of the materials due their well-known resolution limits.

The inner structure for three different MS-MP prototypes (A–C) prepared under the conditions summarized in Table 1 was characterized by *N*_2_ adsorption–desorption experiments (Fig. 1D). The data revealed very high specific surface areas for prototypes A and B (>200 m^2^/g), but even more for prototype C (350 m^2^/g) (Table 2). The porosity of all samples was high (>50%), and the mean pore diameter was ∼12 nm for prototypes A and B, and ∼6 nm for prototype C. These pore sizes were significantly smaller than MS-MPs prepared in our previous work (Pastor et al., 2011), a result of the different preparation conditions. Due to their tighter internal structure, we expected that the MS-MPs obtained in this work would be more suitable for the sustained release of proteins.

**Table 2:** Characteristics of the different mesoporous silicon microparticle prototypes. Data represent means ± S.D., *n* = 3.

Prototype	Specific surface (m^2^/g)	Porosity (%)	Pore diameter (nm)
A	210.2 ± 13	72 ± 6	11.4 ± 0.7
B	224.9 ± 16	53 ± 8	12.4 ± 3
C	350.8 ± 21	60 ± 5	5.8 ± 0.4

Due to the limited number of prototypes studied and the important difference in parameters observed, it is difficult to draw unequivocal conclusions on the relationships between the MS-MPs preparation parameters (Table 1) and the resulting carrier properties (Table 2). Still, under the tested preparation conditions, there is a positive correlation between the current density and the specific surface area. Also, an inverse correlation between the applied current density and the mean pore diameter can be noted, although the doping level of Si wafer might play a dominant role in this correlation. Globally, the study confirms the possibility to prepare MS-MPs with controllable mesoporous inner structures by the electrochemical method.

### Protein loading in MS-MPs

After characterization of the different MS-MP prototypes, we studied how these systems are capable of loading and releasing two proteins, aCT and BMP7. The zymogen aCT was selected as a model protein for screening studies since it has very similar physicochemical properties (pI and Mw) to BMP7 (see data on ‘Materials’), and we have previously observed good correlation between encapsulation of both proteins (Reguera-Nuñez et al., 2014). ACT is a zymogen physiologically activated by the gut’s endopeptidases, and does not activate under the conditions of the loading procedures and release tests applied in this work. For protein loading in this work we decided to work under forcing conditions, and we evaporated a protein solution in the presence of the MS-MPs at 37 °C. This method has the main advantage of forcing protein encapsulation, which can be assumed to be close to 100%. Because MS-MPs cannot be degraded without harming the loaded protein, we were unable to quantify the loaded proteins. However, from the final release point of our release studies (see ‘Pore size can control the release of a model protein (aCT) from MS-MPs’ and ‘MS-MPs can achieve a 2-week sustained release of antigenically active BMP7’), we can conclude that >75% of aCT was loaded in all preparations, and >60% of BMP7.

When using this loading method, the mechanisms that drive protein loading would be capillary forces and adsorption from a continually concentrating solution (Karlsson et al., 2003). Other possible mechanisms would be electrostatic interactions; after thermal oxidation the MS-MPs surface bears a negative charge as the silicon oxides cover the entire porous network (Zangooie, Bjorklund & Arwin, 1998). This might affect the loading and release of cationic proteins such as aCT and BMP7. Under the tested conditions, the final protein payloads per mg of the carrier were 60 µg for aCT and 0.1 µg for BMP7.

### Pore size can control the release of a model protein (aCT) from MS-MPs

**Figure 2:**
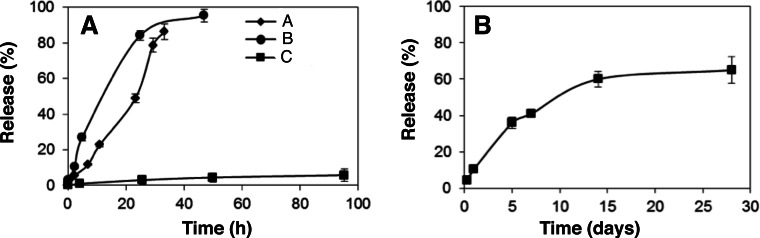
*In vitro* release profile of (A) *α*-chymotrypsinogen and (B) BMP7 from MS-MPs prepared by the electrochemical method. Data represent means ± S.D., *n* = 3.

The release of loaded aCT from the three MS-MPs prototypes was analyzed *in vitro* (PBS, 37 °C). No burst release was observed for any of the tested prototypes, suggesting that most protein is inside the pores and not adsorbed on the outer MS-MP surface (Fig. 2A). This behavior is in agreement with our previous study on insulin and BSA, where despite of a faster release (<2 h), only a moderate burst effect was observed (∼30%) (Pastor et al., 2011). In the present work, the burst effect was drastically reduced, presumably because of the lower pore size of the carriers, and because of the different procedures for protein loading (solvent evaporation vs. adsorption equilibrium).

The MS-MPs investigated in this work were able to control protein release for longer periods of time than the carriers previously reported by us (Pastor et al., 2011): for prototypes A and B a ∼100% release was achieved in 30–40 h after incubation at 37 °C in PBS. Prototype C showed even more sustained kinetics, with high retention of aCT after 96 h (Fig. 2A). However, after 2-weeks, sample C had released 77.2% ± 4.2 (*n* = 3) of the loaded aCT. The slower release should be associated with the nanostructure of the carriers, mainly to their pore size. Mean pore size was <15 nm for all prototypes studied here, and 33 nm in our previous work. Prototype C possesses pores with a mean size of ∼6 nm, half of those of prototypes A and B, and similar to the radius of gyration of aCT, 1.76 nm (Perkins et al., 1993). As observed in other systems (Santos, Radin & Ducheyne, 1999; Peppas et al., 2000; Sandor et al., 2001), when the drug’s radius of gyration is about the size of pores in the matrix, diffusion might be hindered, and more sustained release kinetics achieved. When comparing the different prototypes studied in this work, particle inner structure seems to be the critical factor modulating different release kinetics.

When comparing the performance of the MS-MP prototypes from this work with those of our previous work (Pastor et al., 2011), two additional factors need to be considered. First, the effect of the chemical differences of the proteins tested. Insulin and BSA, used before, both bear negative charges in PBS, and therefore, their attachment to MS-MPs surfaced by adsoption shoud be driven mostly by hydrophobic interactions. On the other hand, aCT is positive in PBS, and therefore, ionic interactions with the silicon oxide on the surface of MS-MPs can be important to explain protein adsorption/desorption. Another parameter that could have some limited influence on protein release is the average particle dimensions, which was 33 µm in our previous work, and is 90 µm here (Pastor et al., 2011). Particle dimension will influence the diffusion length within the carrier for the protein. Recently, a new production method yielding planar mesoporous silicon microparticles with a controlled thicknesses, porosity and pore sizes has been reported (Makushok, Matveyeva & Pastor, 2012). These new kind of materials might be interesting for release mechanism studies, since their lateral dimensions, perpendicular to the pore axis, will play no important role in the release process.

### MS-MPs can achieve a 2-week sustained release of antigenically active BMP7

Based on promising data obtained with aCT protein, we tested MS-MP prototype C for the controlled release of a therapeutic protein: BMP7. This protein is approved by FDA and other regulatory agencies for orthopedic applications (OP-1 Putty and OP-1 Implant; Stryker, Kalamazoo, Michigan, US), and it is delivered through a collagen sponge with limited controlled release properties. This limited controlled release has been linked to most of the treatment undesirable effects (Lane, 2001). MS-MPs were loaded with BMP7 as described in ‘Protein loading in MS-MPs’, and the release kinetics of the protein was analyzed. Consistent with the data obtained with aCT, a 2-week sustained release was achieved (Fig. 2B). Once again, the release kinetics was characterized by low burst (<10%), and by a sustained release profile for at least 14 days. Maximum release observed over the experiment (28 days) was ∼70%. Significantly, the quantification of BMP7 in the supernatant was performed by ELISA, and thus it guarantees the presence of the protein in its antigenically-active form upon release. While antigenic activity is not a final proof of biological activity, previous studies from our group using the same ELISA kit have found a relation between antigenic BMP7 and bioactive protein in a glioblastoma cancer stem cell model (Reguera-Nuñez et al., 2014).

The release profile was fitted to zero-order, first-order, Higuchi and to the Kosmeyer–Peppas models (Wizard—Statistics, Visualization, Data Analysis, Predictive Modeling, version 1.4; Evan Miller©, US). Fitting to the first-order and Higuchi models was adequate (*p* < 0.008 and *p* < 0.002, respectively), but the best fit was achieved with the Kosmeyer–Peppas model (BMP released% =10.4 ⋅ *t* (days)^0.64^, *p* < 0.001). The Kosmeyer–Peppas model is effective to describe release systems where release kinetics might depend on several factors. The diffusional exponent (*n* = 0.64) indicates a process of anomalous diffusion (Korsmeyer et al., 1983; Peppas, 1985).

The similarities between aCT and BMP7 release kinetics reflect their similar physicochemical properties. Indeed, BMP7 has a radius of gyration ∼3.5 nm (by analogy with other BMPs, (Berry et al., 2006)) just slightly larger than aCT. It has also a basic isoelectric point (8.1) close to that of aCT (9.5). These similarities result in consistent profiles for both proteins, and suggest the robustness of the delivery technology.

In summary, we have achieved sustained release of BMP7 for at least two weeks by using electrochemically synthesized MS-MPs. A preparation technology for the whole therapeutic system is convenient, since both components, protein solution and pre-formed empty MS-MPs, can be integrated together in an extemporaneous process. Due to the recently reported osteointegration properties of the MS-MP carrier itself (Sun et al., 2007), one of the immediate promising applications of this system would be in the bone regeneration area.

## Conclusions

Mesoporous silicon microparticles with controlled inner structure (pore size) can be prepared by an electrochemical method, and loaded with proteins by simple adsorption and solvent evaporation. Under optimized electrochemical conditions, these microparticles present a nanostructure with pore sizes below 10 nm, and this small pore size is critical to provide sustained protein release over several days. The medical potential of the electrochemically synthesized mesoporous silicon microparticles is suggested by the two weeks sustained release profile of the osteogenic factor BMP7.

## Supplemental Information

10.7717/peerj.1277/supp-1 Figure S1 Mesoporous silicon Microparticles micrograph (Fig. 1A) Original scanning electron microscopy image of the mesoporous silicon microparticles (Fig. 1A in the publication).

10.7717/peerj.1277/supp-2 Figure S2 Surface of mesopous silicon microparticles (Fig. 1C) Original scanning electron microscopy image of the surface of mesoporous silicon microparticles (Fig. 1C in the publication).

10.7717/peerj.1277/supp-3 Supplemental Information 1 Distributive data for particle size analysis Excel data sheet showing the particle class interval (in µm) and the total volume of all the particles as calculated after analysis with ImageJ. The data was used to generate Fig. 1B. The distributions are calculated for Samples A, B and C.

10.7717/peerj.1277/supp-4 Supplemental Information 2 Nitrogen adsorption isotherms Excel data sheet. N2 adsorbed volume (cm^3^/g) vs. the pressure/saturation pressure ratio for Samples A, B, C. This data was used to prepare Fig. 1D and the calculations for Table 1.

10.7717/peerj.1277/supp-5 Supplemental Information 3 chymotripsinogen release data Excel data sheet. Percentage of chymotripsinogen released over time at 37 °C in PBS buffer for Samples A, B and C (60 µg per mg of microparticle was considered 100%). Data points for each replicate. Time expressed in days.

10.7717/peerj.1277/supp-6 Supplemental Information 4 BMP7 release data Excel data sheet. Percentage of BMP7 released over time at 37 °C in PBS buffer for Sample C (0.1 µg per mg of microparticle was considered 100%). Data points for each replicate. Time was expressed in days.
