# Supplementary figures and images for: Pore size is a critical parameter for obtaining sustained protein release from electrochemically synthesized mesoporous silicon microparticles
Source: PeerJ. 2015 Oct 6;3:e1277. doi: 10.7717/peerj.1277 (PMC4636406; doi:10.7717/peerj.1277)

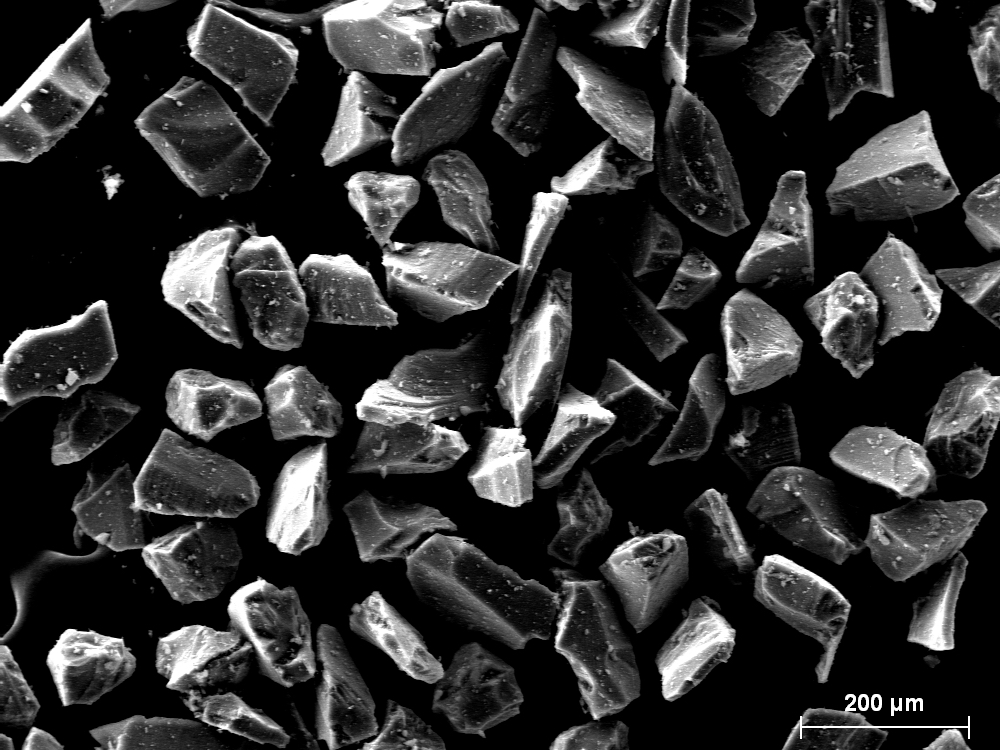

Supplement: Figure S1 — Original scanning electron microscopy image of the mesoporous silicon microparticles (Fig. 1A in the publication). [file peerj-03-1277-s001.jpg]

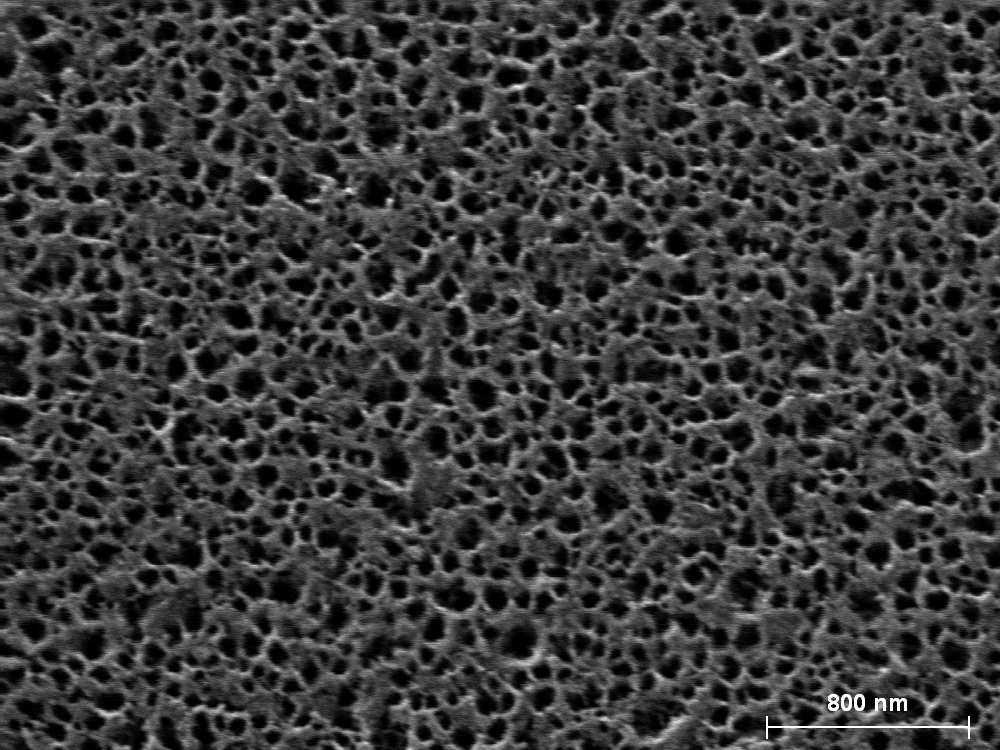

Supplement: Figure S2 — Original scanning electron microscopy image of the surface of mesoporous silicon microparticles (Fig. 1C in the publication). [file peerj-03-1277-s002.jpg]
